# Association between SII and markers of liver injury: A cross-sectional study from the NHANES (2017–2020)

**DOI:** 10.1371/journal.pone.0303398

**Published:** 2024-07-25

**Authors:** Xu-Feng Zhang, Yu-Yan Qin

**Affiliations:** 1 Department of Hepatobiliary Surgery, People’s Hospital of Longhua, Shenzhen, China; 2 Department of General Medicine, People’s Hospital of Longhua, Shenzhen, China; University of Salamanca/University Hospital of Salamanca, SPAIN

## Abstract

**Introduction:**

A novel indicator of inflammation is the systemic immune-inflammation index (SII), and liver dysfunction is linked to the advancement of inflammation. In light of this, this study aims to look into any potential connections between SII and markers of liver injury.

**Methods:**

A cross-sectional study was conducted using the National Health and Nutrition Examination (NHANES) dataset for 2017–2020. The linear relationship between SII and markers of liver injury was examined using multiple linear regression models. Examining threshold effects and fitted smoothed curves were utilized to describe nonlinear connections.

**Results:**

A total of 8213 adults aged 18–80 years participated in this population-based study. In the fully adjusted model, SII maintained a negative association with ALT(β = -0.003, 95%CI:-0.005, -0.002, P<0.00001), AST(β = -0.004, 95% CI:-0.005, -0.002, P<0.00001), and GGT(β = -0.004, 95% CI:-0.007, -0.000, P = 0.03791) and a positive association with ALP (β = 0.005, 95% CI:0.003, 0.007, P<0.00001). In subgroup analyses, it was found that SII remained negatively correlated with ALT, AST and GGT in gender, age and body mass index. SII was positively correlated with ALP at BMI≥25(kg/m2)(β = 0.005, 95% CI:0.003, 0.008, P = 0.00001), and was negatively correlated with ALT(β = -0.004, 95% CI:-0.005, -0.002, P<0.00001), AST(β = -0.004, 95% CI:-0.005, -0.003, P<0.00001) and GGT(β = -0.004, 95% CI:-0.008, -0.000, P = 0.02703) at BMI≥25, whereas no significant correlation was observed at BMI<25 (all P-values>0.05). Furthermore, the association between SII and markers of liver injury was nonlinear. By using a two-stage linear regression model for analysis, a U-shaped relationship was found to exist between SII and ALT with a turning point of 818.40(1,000 cells/μl). The inflection points of SII with AST and GGT were 451.20 (1,000 cells/μl) and 443.33 (1,000 cells/μl), respectively, and no significant inflection point with ALP was observed. Interaction tests demonstrated that SII correlation with ALT, AST, ALP, and GGT was not significantly different between strata (all p for interaction>0.05).

**Conclusions:**

The research findings suggested that there was a negative correlation between SII and ALT, AST and GGT, and a positive correlation with ALP. However, larger prospective investigations are still greatly needed to confirm the findings.

## Introduction

As a comprehensive new inflammatory biomarker, SII has been suggested to be a valuable gauge of inflammation in human body issues and systemic immune health by mounting data [1–3]. Some studies have shown that SII has predictive value in colorectal and postoperative hepatocellular carcinoma [1, 2]. Hu et al. emphasized that in patients with hepatocellular carcinoma, high SII scores (≥330) were linked to poor postoperative outcomes [2]. Furthermore, there is a connection between systemic inflammatory response and hepatic dysfunction [4].

According to statistics, 500 million individuals worldwide suffer from chronic liver disease in a given year. As the condition worsens, patients have consequences from portal hypertension and hepatocellular dysfunction, raising the risk of liver-related morbidity and death. About 44,000 fatalities in the US and 20,000 deaths worldwide are attributed to chronic liver disease and cirrhosis, which also remarkably increases the cost of healthcare and causes a large burden of disability [5]. Non-alcoholic fatty liver disease (NAFLD) is the most common chronic liver disease in the world and one of the main causes of severe liver disease [6–9]. It is linked to insulin resistance, hypertension, atherosclerosis, obesity, and dyslipidemia and is thought to be the hepatic manifestation of metabolic syndrome. It is brought on by hepatocyte fat buildup, which triggers an inflammatory response and oxidative damage. Some studies have revealed that nutritional supplements, Chlorella vulgaris(CV) may reduce oxidative stress and inflammation to some extent and also exhibit the potential to improve body composition and enhance physical functioning [10, 11]. Hepatic impairment of varied degrees is frequently present at the outset of liver disease, and liver function tests (LFTs) are frequently performed to measure liver impairment [12]. Alkaline phosphatase (ALP) [13], aspartate aminotransferase(AST), gamma-glutamyl transferase(GGT) and alanine aminotransferase(ALT) are common indicators of liver dysfunction. For the purpose of effective illness control and prevention in the future, factors that contribute to liver function decline must be scientifically and systematically recognized and conveyed. However, the relationship between different degrees of SII and markers of liver injury is currently not clear.

In this regard, a population-based cross-sectional study examined the association between the systemic immune inflammatory index (SII) and markers of liver injury in adult National Health and Nutrition Examination Survey (NHANES) participants.

## Methods

### Study design and population

**Fig 1:**
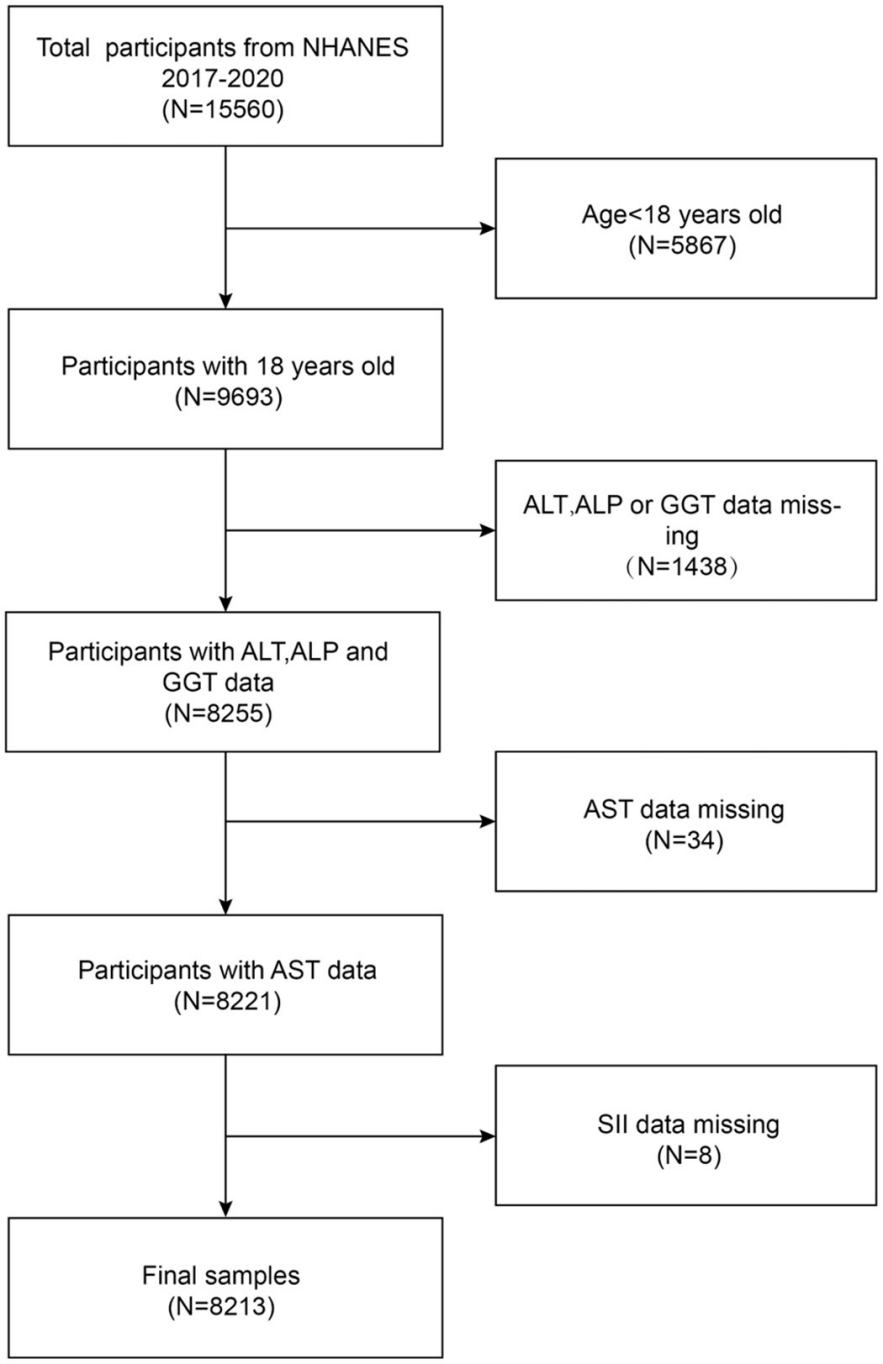
Flowchart of participant selection.

All NHANES participants are required to provide written informed permission by clearance from the National Center for Health Statistics Institutional Review Board. The NHANES data collection is accessible to the public. By selecting a representative sample of the U.S. population, the National Center for Health Statistics (NCHS) carried out a population-based nationwide cross-sectional research called NHANES to comprehensively look into both nutrition and health status in the U.S. Therefore, the NHANES data was adopted in this study [14]. The sample was representative since the investigation was carried out in two-year cycles utilizing intricate multistage stratified probability sampling. The NCHS study Ethics Review Board authorized all NHANES study procedures, and informed consent forms were signed by survey respondents, survey parents, and/or survey legal guardians of persons under the age of 16. All detailed NHANES study designs and data are publicly available at www.cdc.gov/nchs/nhanes/. An abundance of data about the nutrition and health of the US population at large is provided by the NHANES, a representative survey of the country’s population that employs a complex, multistage, and probabilistic sampling process [15]. The 2017–2020 consecutive cycles of the US NHANES dataset were chosen for this study. Initially, 15,560 participants were recruited, and participants younger than 18 years of age (n = 5867) were excluded, thus missing ALT, ALP, GGT, and AST lab data (n = 1472) and missing SII data (n = 8). Ultimately, a total of 8213 participants were included in this research. This investigation was approved by the National Health and Nutrition Examination Survey Ethical Review Board, without requiring external ethical approval. Fig 1 illustrates the sample selection flowchart.

### Exposure variables

SII is based on lymphocyte, neutrophil, and platelet counts expressed as ×10^3^ cells/μl are measured using automated hematology analysis equipment. The following formula is used to calculate SII: (platelet count×neutrophil count)/lymphocyte count [16].

### Outcome variables

Liver injury markers mainly include alanine aminotransferase(ALT), aspartate aminotransferase(AST), gamma-glutamyl transferase (GGT), and alkaline phosphatase (ALP) [13].

#### Covariates

The survey included covariates that may influence the relationship between SII and markers of liver injury(ALT, AST, ALP, and GGT). Demographic parameters en-compassed age(years), gender(male/female), race(Mexican American/other Hispanic/non-Hispanic white/non-Hispanic black/other race), education level(less than high school, high school or general education development/higher than high school), tobacco use, BMI (BMI, kg/m^2^), alcohol use(yes/no), moderate exercise(yes/no), protein intake(gm), energy intake(kcal), dietary fiber intake(gm) and the household income to poverty ratio(PIR). Total bilirubin and total protein were incorporated into biochemical characteristics. All detailed measurement procedures for these variables are publicly available at www.cdc.gov/nchs/nhanes/.

### Statistical analyses

Given the intricacy of the sample, a weighted analysis was conducted in compliance with NHANES guidelines. For statistical research, R (version 4.1.3) and EmpowerStats (version 2.0) were utilized for graphing and statistical computations. The quartile groupings of ALT, AST, ALP, and GGT with SII were employed to statistically characterize the research population’s baseline table, and continuous variables were characterized by weighted linear regression models with mean positive or negative standard deviation(SD). Multivariate linear regression analysis was used to determine beta values and 95% confidence intervals between SII and ALT, AST, ALP, and GGT. The multivariate tests were constructed based on three models: Model 1 shows no variable modification; Model 2 shows age, gender, and race adjustments; and Model 3 shows all covariate adjustments. Simultaneous variable adjustment and smoothed curve fitting were carried out. The inflection points between SII and ALT, AST, ALP, and GGT were examined by a threshold effect analysis model. The age, gender, and BMI subgroups were then subjected to the same statistical research techniques as previously mentioned, and an interaction term was included to test for heterogeneity of associations between subgroups (p<0.05 being regarded statistically significant). In addition, a weighting technique was used to lessen the dataset’s notable volatility.

## Results

### Baseline characteristics of participants

Individuals’ weighted attributes were divided into quartiles according to their SII level (Q1: 10.73 < SII< 315.65; Q2: 316 < SII < 450.46; Q3:450.5 < SII < 635.93; Q4: 635.95 < SII< 4887.75), as shown in Table 1. This research comprised 8213 individuals between the ages of 18 and 80, with a mean age of 47.58±17.78 years, 48.42% males and 51.58% females. There were statistically significant differences in different quartiles of SII in terms of age, gender, ethnicity, educational attainment, poverty-to-income ratio, BMI, ALT, AST, ALP, GGT, bilirubin, protein intake, energy intake, dietary fiber intake, and total protein (all p-values<0.05). In contrast to the group with the lowest SII values, individuals with the highest SII values were likely to be older white women, with higher BMI and ALP, as well as lower AST, ALT, GGT, total bilirubin, total protein, protein intake, energy intake, dietary fiber intake, and income poverty ratio, consistent with previous reports in the literature that pursuit of a healthy lifestyle is associated with lower SII [17]. In addition, there were no statistically significant differences in appropriate exercise, smoking, and alcohol use(all p-values>0.05).

**Table 1:** Weighted baseline characteristics of the participants according to different systemic immune-inflammatory index (SII).

SII	Q1	Q2	Q3	Q4	*P*-value
	10.73 < SII < 315.65	316 < SII < 450.46	450.5 < SII < 635.93	635.95 < SII < 4887.75	
Age (years)	46.81 ± 17.48	46.37 ± 17.48	47.77 ± 18.06	49.15 ± 17.91	<0.0001
Gender (%)					<0.0001
Men	57.04	51.41	45.04	42.02	
Women	42.96	48.59	54.96	57.98	
Race/Ethnicity (%)					<0.0001
Mexican American	8.54	10.26	8.83	7.61	
Other Hispanic	7.18	8.30	7.15	8.01	
Non-Hispanic white	52.62	62.51	65.75	68.81	
Non-Hispanic black	19.48	9.91	8.62	6.55	
Other races	12.18	9.03	9.65	9.02	
Education lever (%)					0.0013
Less than 9^th^ grade	4.02	3.85	3.89	3.24	
9–11th grade	6.18	7.49	7.55	7.68	
High school graduate	26.58	25.14	26.97	28.41	
Associate degree	27.74	30.38	31.40	32.05	
College or above	35.40	33.13	29.95	28.57	
Moderate activities (%)					0.1048
Yes	50.05	49.98	46.16	49.26	
No	49.83	49.99	53.82	50.69	
Smoked at least 100 cigarettes					0.0905
Yes	41.09	39.75	42.10	44.30	
No	58.85	60.25	57.88	55.66	
Income to poverty ratio	3.11 ± 1.68	3.21 ± 1.65	3.19 ± 1.61	2.98 ± 1.63	<0.0001
BMI (kg/m^2^)	28.21 ± 6.27	29.24 ± 6.74	30.28 ± 7.12	30.92 ± 8.33	<0.0001
Alcohol consumption					0.0504
Yes	91.64	92.67	93.92	93.24	
No	8.36	7.33	6.08	6.76	
Energy(kcal)	2122.91 ± 857.30	2059.96 ±769.92	2062.62 ±811.60	2042.62 ±804.99	0.0391
Protein(gm)	81.66 ± 35.22	81.49 ±34.44	79.53 ±33.91	76.44 ±33.36	<0.0001
Dietary fiber(gm)	17.35±9.25	16.38±8.47	16.03±9.54	15.54±8.22	<0.0001
ALT, IU/L	23.56 ± 20.54	23.06 ± 15.70	22.42 ± 19.22	21.66 ± 16.56	0.0057
AST, IU/L	24.09 ± 17.52	21.83 ± 12.06	21.14 ± 11.38	20.71 ± 11.66	<0.0001
ALP, IU/L	73.55 ± 25.51	73.32 ± 24.74	75.92 ± 23.91	79.29 ± 26.25	<0.0001
GGT, IU/L	32.23 ± 59.82	27.93 ± 39.10	28.08 ± 34.77	29.22 ± 35.12	0.0065
Total Bilirubin (umol/L)	8.56 ± 5.17	8.21 ± 5.70	7.85 ± 4.50	7.65 ± 4.55	<0.0001
Total Protein (g/L)	71.24 ± 4.48	71.05 ± 4.24	70.79 ± 4.23	70.93 ± 4.51	0.0108
SII(1,000 cells/μl)	241.80 ± 55.36	383.95 ± 37.91	534.90 ± 53.74	919.97 ± 416.01	<0.0001

Mean ± SD for continuous variables: the p-value was calculated by a weighted linear regression model. % for categorical variables: the p-value was calculated by a weighted chi-square test. BMI, body mass index; ALT, alanine aminotransferase; AST, aspartate aminotransferase; ALP, alkaline phosphatase; GGT, gamma-glutamyl transferase; SII, systemic immune-inflammation index.

### Association between SII and markers of liver injury

Table 2 provides a summary of the multifactorial regression analysis results. In the uncorrected model, SII was negatively correlated with ALT (β = -0.003, 95% CI:-0.004, -0.002, P<0.00001) and AST (β = -0.003, 95% CI:-0.004, -0.003, P<0.00001), and SII was positively correlated with ALP (β = 0.007, 95% CI:0.005, 0.009, P<0.00001). In adjusted model 2, ALT and SII were positively correlated. (β = -0.002,95% CI:-0.004, -0.001, P = 0.00011) and AST(β = -0.003, 95% CI:-0.004, 0.002, P<0.00001). In adjusted model 3, SII was positively associated with ALT (β = -0.003, 95% CI:-0.005, -0.002, P<0.00001), and remained negatively correlated with AST (β = -0.004, 95% CI:-0.005, -0.002, P<0.00001). In adjusted models 2 and 3, SII remained positively correlated with ALP (β = 0.007, 95% CI:0.005, 0.009, P<0.00001; β = 0.005, 95% CI:0.003, 0.007, P<0.00001). SII and GGT were not significantly correlated in the unadjusted model (β = -0.003, 95% CI:-0.006, -0.000, P = 0.06859) and adjusted model 2(β = -0.001, 95% CI:-0.004, -0.002, P = 0.51127) (all p-values>0.05), and in the adjusted model 3, SII and GGT were negatively correlated (β = -0.004, 95% CI-0.007, -0.000, P = 0.03791). Further, SII was changed for sensitivity analysis from a continuous variable to a categorical variable (quartiles). In terms of ALT, AST and GGT, the highest SII quartile obtained a lower ALT value of 3.82 units compared with the lowest SII quartile (β = -3.825,95% CI:-5.074, -2.577, P for trend<0.00001), AST values were 3.86 units lower (β = -3.867, 95% CI:-4.874, -2.861, P for trend<0.00001), and GGT values were 4.66 units lower (β = -4.664, 95% CI:-8.054, 0.25, P for trend = 0.00826). In terms of ALP, the highest SII quartile achieved ALP values that were 3.74 units higher than the lowest quartile (β = 3.742, 95% CI:1.703, 5.782, P for trend = 0.00026).

**Table 2:** Association between SII and markers of liver injury.

	Model 1 b (95% CI)	Model 2 b (95% CI)	Model 3 b (95% CI)
	*P* value	*P* value	*P* value
ALTK, IU/L	-0.003 (-0.004, -0.002) <0.00001	-0.002 (-0.004, -0.001) 0.00011	-0.003 (-0.005, -0.002) <0.00001
AST, IU/L	-0.003 (-0.004, -0.003) <0.00001	-0.003 (-0.004, -0.002) <0.00001	-0.004 (-0.005, -0.002) <0.00001
ALP, IU/L	0.007 (0.005, 0.009) <0.00001	0.007 (0.005, 0.009) <0.00001	0.005 (0.003, 0.007) 0.00002
GGT, IU/L	-0.003 (-0.006, 0.000) 0.06859	-0.001 (-0.004, 0.002) 0.51127	-0.004(-0.007, 0.000) 0.03791
ALT			
Quintiles of SII			
Q1	Reference	Reference	Reference
Q2	-0.337 (-1.495, 0.820) 0.56773	-0.716 (-1.849, 0.417) 0.21563	-0.910 (-2.123, 0.304) 0.14192
Q3	-1.473 (-2.630, -0.315) 0.01269	-1.495 (-2.635, -0.354) 0.01024	-2.229 (-3.450, -1.008) 0.00035
Q4	-2.468 (-3.626, -1.310) 0.00003	-2.187 (-3.337, -1.036) 0.00020	-3.825 (-5.074, -2.577) <0.00001
*P* for trend	<0.0001	0.00011	<0.0001
AST			
Quintiles of SII			
Q1	Reference	Reference	Reference
Q2	-1.817 (-2.707, -0.926) 0.00006	-1.896 (-2.787, -1.006) 0.00003	-1.775 (-2.754, -0.797) 0.00038
Q3	-2.974 (-3.864, -2.083) <0.00001	-2.906 (-3.802, -2.009) <0.00001	-2.759 (-3.743, -1.775) <0.00001
Q4	-3.598 (-4.489, -2.708) <0.00001	-3.426 (-4.330, -2.521) <0.00001	-3.867(-4.874, -2.861) <0.00001
*P* for trend	<0.00001	<0.00001	<0.00001
ALP			
Quintiles of SII			
Q1	Reference	Reference	Reference
Q2	0.929 (-0.724, 2.582) 0.27057	1.171 (-0.467, 2.809) 0.16110	0.942 (-1.041, 2.925) 0.35198
Q3	2.531 (0.877, 4.184) 0.00271	2.802 (1.153, 4.451) 0.00087	0.960 (-1.035, 2.955) 0.34571
Q4	5.986 (4.333, 7.639) <0.00001	6.229 (4.565, 7.892) <0.00001	3.742 (1.703, 5.782) 0.00033
*P* for trend	<0.00001	<0.00001	0.00026
GGT			
Quintiles of SII			
Q1	Reference	Reference	Reference
Q2	-4.036 (-7.170, -0.901) 0.01163	-2.883 (-6.018, 0.252) 0.07150	-2.476 (-5.771, 0.820) 0.14100
Q3	-5.735 (-8.870, -2.600) 0.00034	-3.818 (-6.974, -0.663) 0.01773	-3.968(-7.283, -0.652) 0.01903
Q4	-4.535 (-7.670, -1.400) 0.00459	-2.102 (-5.285, 1.081) 0.19560	-4.664(-8.054, 0.257) 0.00703
*P* for trend	0.01024	0.32074	0.00826

In Model 1, no covariates were adjusted. In Model 2, age, gender, and race were adjusted. In Model 3, age, gender, race, education level, moderate activities, smoking at least 100 cigarettes, poverty-to-income ratio, BMI, total bilirubin, total protein, protein intake, energy intake, dietary fiber intake, and alcohol consumption were adjusted. 95%CI, 95% confidence interval; SII, systemic immunity-inflammation index; ALT, alanine aminotransferase; AST, aspartate aminotransferse; ALP, alkaline phosphatase; GGT, gamma-glutamyl transferase. p<0.05 was considered statistically significant.

### Subgroup analysis for the association between SII and markers of liver injury

Subgroup analyses using age, gender, and BMI stratification were carried out. Stratified weighted multiple regression analyses were performed to investigate the relationship between SII and indicators of liver injury in various population contexts and look for interactions (Table 3). The results showed that SII remained was negatively correlated with ALT, AST and GGT in gender, age, and body mass index, SII was positively correlated with ALP at BMI ≥25 (β = 0.005, 95% CI:0.003, 0.008, P = 0.00001), and SII was negatively correlated with ALT (β = -0.004, 95% C-I:-0.005, -0.002, P<0.00001), AST(β = -0.004, 95% CI:-0.005, -0.003, P<0.00001) and GGT(β = -0.004, 95% CI:-0.008, -0.000, P = 0.02703) at BMI≥25, whereas they were not significantly correlated at BMI <25 (all P values> 0.05). In addition, the interaction tests manifested that SII correlations with ALT, AST, ALP, and GGT were not significantly different between strata, indicating the non-existence of significant dependence of gender, age, and BMI on the negative correlation of SII with ALT, AST, and GGT, and the positive correlation of SII with ALP (all p for interaction>0.05).

**Table 3:** Subgroup analysis stratified by different variables, weighted.

	Model 1 b (95% CI)	Model 2 b (95% CI)	Model 3 b (95% CI)	*P* for interaction
	*P* value	*P* value	*P* value	
Stratified by gender				
ALT				0.8950
Men	-0.003(-0.005,-0.001) 0.00916	-0.002(-0.004,-0.000) 0.02953	-0.003(-0.005,-0.001) 0.00517	
Women	-0.002(-0.003,-0.000) 0.02113	-0.002(-0.003,-0.001) 0.00416	-0.003(-0.004,-0.001) 0.00020	
AST				0.6866
Men	-0.004(-0.005,-0.002) <0.00001	-0.003(-0.005,-0.002) 0.00002	-0.004(-0.005,-0.002) 0.00002	
Women	-0.003(-0.004,-0.002) <0.00001	-0.003(-0.004,-0.002) <0.00001	-0.003(-0.004,-0.002) 0.00003	
ALP				0.1651
Men	0.007 (0.005, 0.010) <0.00001	0.007 (0.005, 0.010) <0.00001	0.004 (0.001, 0.007) 0.01228	
Women	0.007 (0.004, 0.009) <0.00001	0.008 (0.005, 0.010) <0.00001	0.007 (0.004, 0.010) <0.00001	
GGT				0.3153
Men	-0.003 (-0.009,0.003) 0.31205	-0.002 (-0.008,0.004) 0.45136	-0.006(-0.012,0.000) 0.05486	
Women	-0.001 (-0.004,0.002) 0.55010	0.000 (-0.003, 0.004) 0.80979	-0.001 (-0.005,0.003) 0.66191	
Stratified by age				
ALT				0.8118
<60 years old	-0.002(-0.004,-0.001) 0.00653	-0.001 (-0.003,0.000) 0.14325	-0.003(-0.005,-0.001) 0.00056	
≥60 years old	-0.003(-0.005,-0.002) 0.00002	-0.003(-0.004,-0.002) 0.00003	-0.003(-0.005,-0.001) 0.00109	
AST				0.8996
<60 years old	-0.004(-0.005,-0.002) <0.00001	-0.003(-0.004,-0.002) 0.00001	-0.003(-0.005,-0.002) <0.00001	
≥60 years old	-0.003(-0.004,-0.002) <0.00001	-0.003(-0.004,-0.002) <0.00001	-0.003(-0.005,-0.001) 0.00024	
ALP				0.7462
<60 years old	0.007 (0.005, 0.010) <0.00001	0.008 (0.006, 0.010) <0.00001	0.005 (0.002, 0.008) 0.00125	
≥60 years old	0.006 (0.003, 0.008) 0.00005	0.006 (0.004, 0.009) <0.00001	0.005 (0.002, 0.009) 0.00125	
GGT				0.5863
<60 years old	-0.004 (-0.009,0.000) 0.07962	-0.001 (-0.006,0.004) 0.72992	-0.004 (-0.009,0.001) 0.08564	
≥60 years old	-0.002 (-0.006,0.003) 0.43958	0.000 (-0.004, 0.005) 0.90711	-0.002 (-0.007,0.003) 0.46666	
Stratified by BMI(kg/m2)				
ALT				0.4588
BMI<25	-0.002 (-0.005,0.000) 0.08099	-0.002 (-0.005,0.000) 0.10546	-0.002(-0.005,-0.000) 0.07795	
BMI≥25	-0.003(-0.005,-0.002) <0.00001	-0.003(-0.004,-0.002) 0.00002	-0.004(-0.005,-0.002) <0.00001	
AST				0.3331
BMI<25	-0.002(-0.004,-0.000) 0.04057	-0.002(-0.004,-0.000) 0.04145	-0.003(-0.005,-0.000) 0.05244	
BMI≥25	-0.004(-0.005,-0.003) <0.00001	-0.004(-0.005,-0.003) <0.00001	-0.004(-0.005,-0.003) <0.00001	
ALP				0.2084
BMI<25	0.005 (0.002, 0.008) 0.00291	0.004 (0.000, 0.007) 0.02404	0.003 (-0.002, 0.007) 0.25519	
BMI≥25	0.008 (0.006, 0.010) <0.00001	0.008 (0.006, 0.010) <0.00001	0.005 (0.003, 0.008) 0.00001	
GGT				0.8178
BMI<25	-0.001 (-0.007,0.005) 0.82752	-0.000 (-0.006,0.006) 0.97353	-0.003 (-0.012,0.006) 0.51075	
BMI≥25	-0.004 (-0.008,0.000) 0.06067	-0.001 (-0.006,0.003) 0.48507	-0.004 (-0.008,-0.000) 0.02703	

In Model 1, no covariates were adjusted. In Model 2, age, gender, and race were adjusted. In Model 3, age, gender, race, education level, moderate activities, smoking at least 100 cigarettes, poverty-to-income ratio, BMI, total bilirubin, total protein, protein intake, energy intake, dietary fiber intake, and alcohol consumption were adjusted. 95% CI, 95% confidence interval; ALT, alanine aminotransferase; AST, aspartate aminotransferase; ALP, alkaline phosphatase; GGT, gamma-glutamyl transferase.

In the subgroup analysis stratified by age, gender and BMI, the model was not adjusted for age, gender, and BMI, respectively.

**Fig 2:**
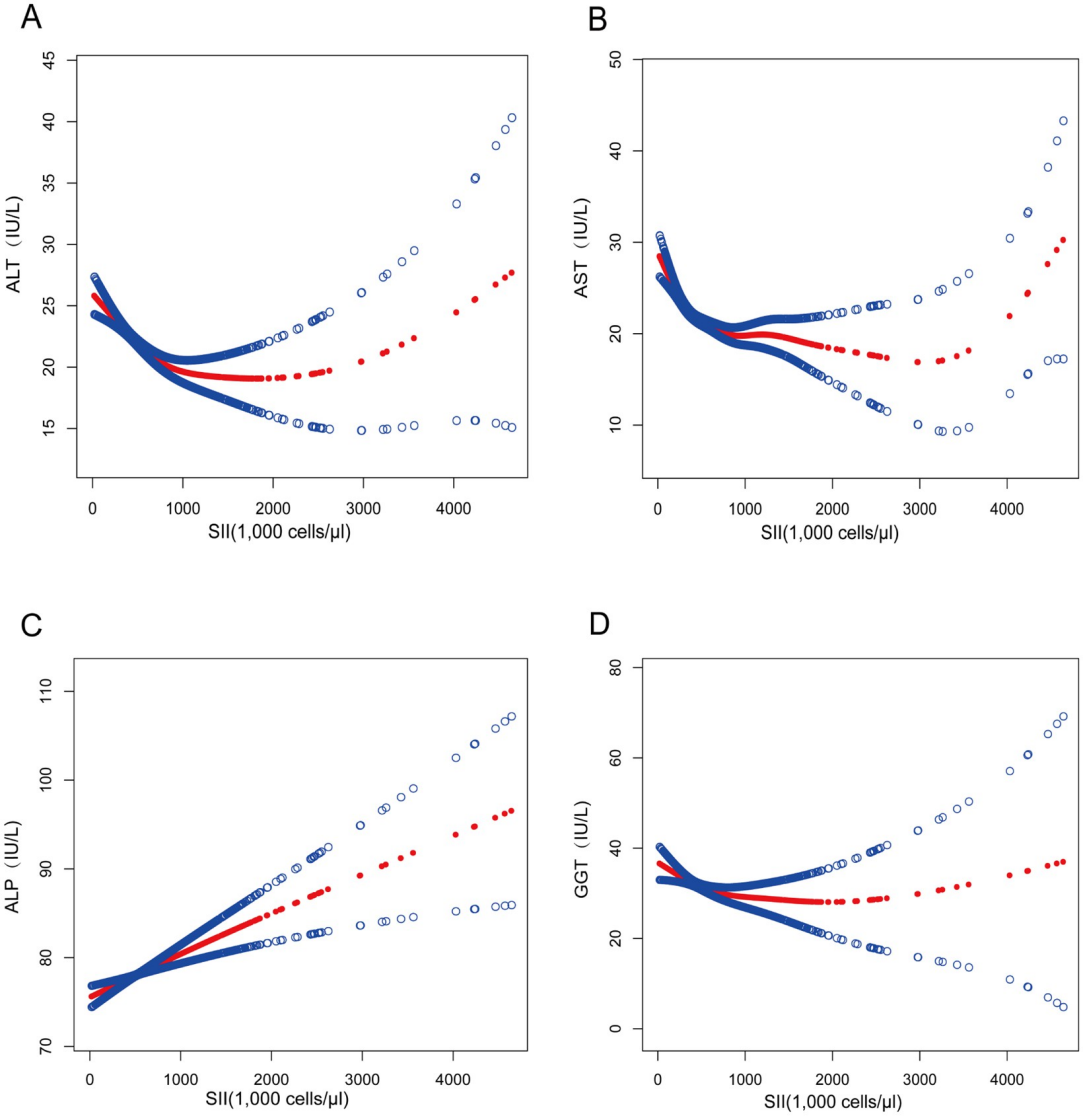
Association between SII and markers of liver injury. The solid red line represents the smooth curve fit between variables. Blue bands represent the 95% confidence interval from the fit.(A)SII, systemic immune-inflammation index; ALT, alanine aminotransferase. (B) SII, systemic immune-inflammation index; AST, aspartate aminotransferase. (C) SII, systemic immune-inflammation index; ALP, alkaline phosphatase. (D) SII, systemic immune-inflammation index; GGT, gamma- glutamyl transferase.

**Fig 3:**
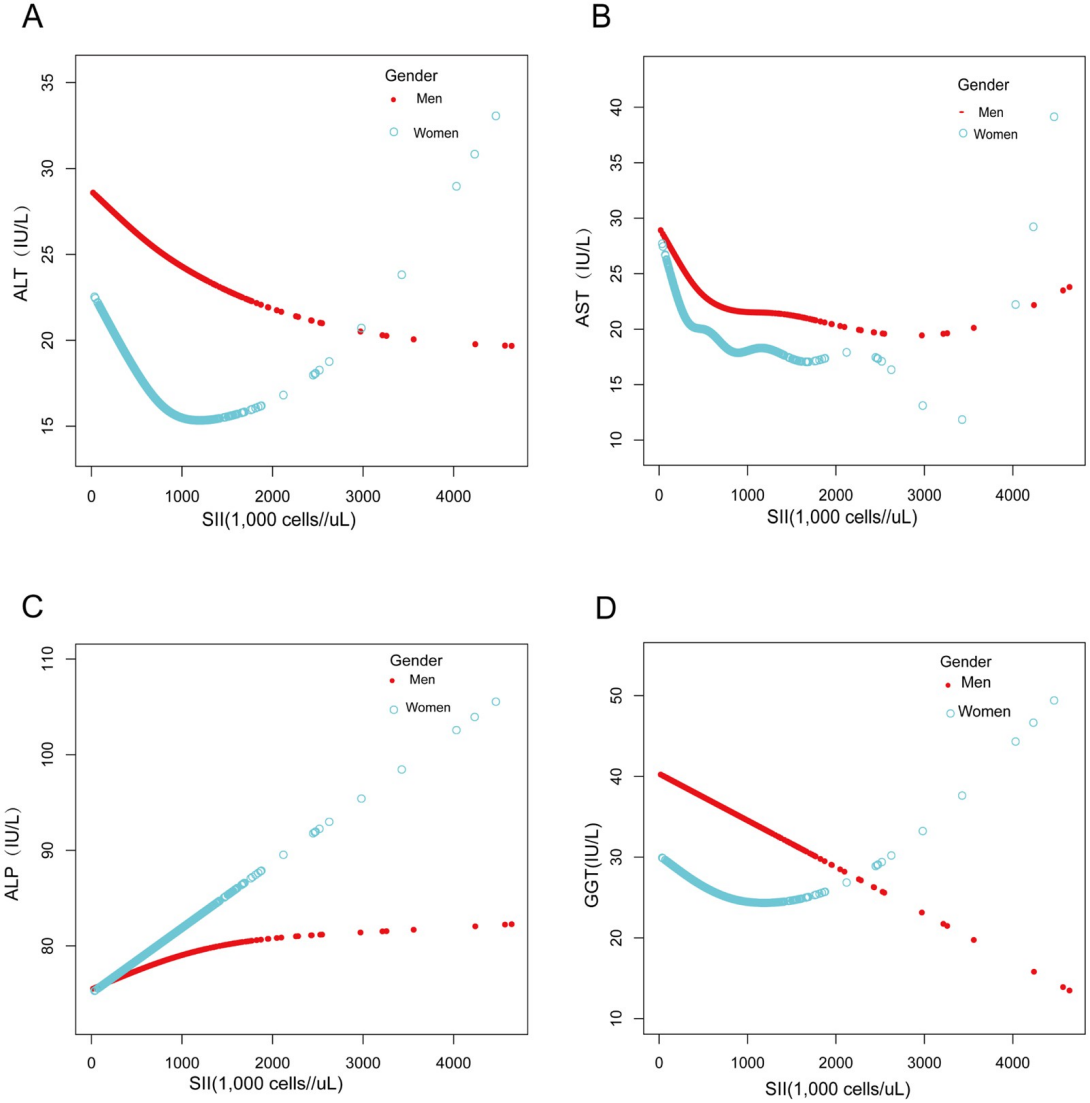
Association between SII and markers of liver injury stratified by gender. (A) SII, systemic immune-inflammation index; ALT, alanine aminotransferase. (B) SII, systemic immune-inflammation index; AST, aspartate aminotransferase. (C) SII, systemic immune-inflammation index; ALP, alkaline phosphatase. (D) SII, systemic immune-inflammation index; GGT, gamma-gluamyl transferase.

Moreover, smoothed curve fitting was adopted to describe the nonlinear associations between SII and markers of liver injury (Figs 2 and 3). The following variables were accordingly adjusted: age, gender, race, education level, poverty rate, BMI, smoking, moderate exercise, total bilirubin, total protein, protein intake, energy intake, dietary fiber intake, and alcohol consumption. A U-curve relationship was discovered between SII and ALT using a two-stage linear regression model with a turning point of 818.40 (1,000 cells/μl), which also appeared in females after stratified analysis by gender, with a turning point of 919.92 (1,000 cells/μl), respectively. A two-stage linear regression model with a turning point of 451.20(1,000 cells/μl) revealed a non-linear relationship between SII and AST. After stratified analysis by gender, males and females also displayed a non-linear relationship. Meanwhile, a two-stage linear regression model with a turning point of 443.33 (1,000 cells/μl) revealed a non-linear relationship between SII and GGT. After stratified analysis by gender, females presented a U-shaped relationship, with a turning point of 195.62(1000 cells/μl). There was not a discernible turning point between SII and ALP (log-likelihood ratio>0.05). (Table 4).

**Table 4:** Threshold effect analysis of SII on markers of liver injury using two-piece wise linear regression model.

**ALT (IU/L)**	**Adjusted b (95% CI) P value**
SII	
Inflection point	818.40
SII<818.40 (1,000 cells/μl)	-0.008 (-0.010,-0.006) <0.0001
SII>818.40(1,000 cells/μl)	0.001 (-0.001, 0.004) 0.2446
Log likelihood ratio	<0.001
Men	
Inflection point	446.00
SII<446.00(1,000 cells/μl)	-0.011 (-0.019,-0.003) 0.0060
SII>446.00(1,000 cells/μl)	-0.001 (-0.004, 0.001) 0.2761
Log likelihood ratio	0.038
Women	
Inflection point	912.92
SII<912.92(1,000 cells/μl)	-0.008 (-0.010,-0.005) <0.0001
SII>912.92(1,000 cells/μl)	0.003 (-0.001, 0.006) 0.0167
Log likelihood ratio	<0.001
**AST (IU/L)**	**Adjusted b (95% CI) P value**
SII	
Inflection point	451.20
SII<451.20(1,000 cells/μl)	-0.015 (-0.019,-0.011) <0.0001
SII>451.20(1,000 cells/μl)	-0.001 (-0.002, 0.000) 0.1190
Log likelihood ratio	<0.001
Men	
Inflection point	453.89
SII<453.89(1,000 cells/μl)	-0.016 (-0.022,-0.011) <0.0001
SII>453.89(1,000 cells/μl)	-0.001 (-0.003, 0.001) 0.3980
Log likelihood ratio	<0.001
Women	
Inflection point	195.62
SII<195.62(1,000 cells/μl)	-0.0916(-0.129,-0.063) <0.0001
SII>195.62(1,000 cells/μl)	-0.002 (-0.003, -0.001) 0.0033
Log likelihood ratio	<0.001
**ALP (IU/L)**	**Adjusted b (95% CI) P value**
SII	
Inflection point	1055.08
SII<1055.08(1,000 cells/μl)	0.006 (0.003, 0.009) <0.0001
SII>1055.08(1,000 cells/μl)	0.002 (-0.002, 0.006) 0.3726
Log likelihood ratio	0.162
Men	
Inflection point	874.59
SII<874.59(1,000 cells/μl)	0.008 (0.003, 0.013) <0.0001
SII>874.59(1,000 cells/μl)	-0.000 (-0.005, 0.005) 0.9645
Log likelihood ratio	0.064
Women	
Inflection point	516.75
SII<516.75(1,000 cells/μl)	0.005(-0.005, 0.014) 0.3203
SII>516.75(1,000 cells/μl)	0.007 (0.004, 0.011) 0.0002
Log likelihood ratio	0.641
**GGT (IU/L)**	**Adjusted b (95% CI) P value**
SII	
Inflection point	443.33
SII<443.33(1,000 cells/μl)	-0.023 (-0.037,-0.009) 0.0011
SII>443.33(1,000 cells/μl)	-0.000 (-0.005, 0.004) 0.9453
Log likelihood ratio	0.005
Men	
Inflection point	459.77
SII<459.77(1,000 cells/μl)	-0.022 (-0.043, -0.001) 0.0372
SII>459.77(1,000 cells/μl)	-0.002 (-0.010, 0.005) 0.5471
Log likelihood ratio	0.107
Women	
Inflection point	195.62
SII<195.62(1,000 cells/μl)	-0.153(-0.256,-0.050) 0.0036
SII>195.62(1,000 cells/μl)	0.000 (-0.004, 0.005) 0.8586
Log likelihood ratio	0.004

Age, gender, race, education level, moderate activities, smoking at least 100 cigarettes, poverty-to-income ratio, BMI, total bilirubin, total protein, protein intake, energy intake, dietary fiber intake, and alcohol consumption were adjusted. 95% CI, 95% confidence interval; ALT, alanine aminotransferase; AST, aspartate aminotransferase; ALP, alkaline phosphatase; GGT, gamma-glutamyl transferase; SII, systemic immune-inflammation index.

## Discussion

According to this cross-sectional study involving 8213 participants, it was found that SII was negatively associated with ALT,AST and GGT, positively associated with ALP. There was no significant dependence of gender, age and BMI on this association. The results of subgroup analyses and interaction tests implied that this association was similar in the population. SII showed a U-shaped relationship with ALT with an inflection point of 818.40 (1,000 cells/μl), and a nonlinear relationship with AST and GGT with inflection points of 451.20 (1,000 cells/μl) and 443.33 (1,000 cells/μl), respectively, and there was no significant inflection point with ALP. The above data indicate that ALT rises when SII exceeds 818.40 (1,000 cells/μl), and AST and GGT increase when it exceeds 451.20 (1,000 cells/μl) and 443.33 (1,000 cells/μl), respectively, and with the rise of SII, ALP also tends to increase to some extent.

To our knowledge, this is the first study to evaluate the association between SII and markers of liver injury. As aforementioned, SII refers to an inflammatory index based on lymphocyte, neutrophil and platelet counts. Bo Hu’s team proposed that SII is an independent postoperative prognostic predictor for hepatocellular carcinoma [2]. This new index combines three independent indices with stronger prognostic predictive ability in cancers such as small-cell lung cancer [18] and esophageal squamous cell carcinoma [19], which is a widely recognized inflammatory indicator. In addition, platelet-to-lymphocyte ratio (PLR) [20], neutrophil-to-lymphocyte ratio(NLR) [21], lymphocyte monocyte ratio(LMR) [22] and lymphocyte-to-monocyte ratio (MLR) [23] based on the number of neutrophils, lymphocytes, monocytes and platelets are biomarkers of the collective immune response, which have been proven to be associated with chronic liver disease [24, 25]. Xie [26] and Song [27] et al. stated that elevated SII levels were associated with hepatic steatosis.

From a comparatively obscure condition to the most prevalent cause of chronic liver disease worldwide, non-alcoholic fatty liver disease (NAFLD) has undergone significant changes [6–9] and numerous epidemiologic studies have demonstrated that inflammation is associated with the progression of NAFLD [28–30], while some studies have shown that NAFLD is accompanied by varying degrees of hepatic impairment [31]. Among them, lipotoxicity, oxidative stress, mitochondrial dysfunction and endoplasmic reticulum stress further lead to hepatocyte injury and death, resulting in hepatic inflammation and immune dysfunction [32]. In this study, liver enzyme levels were considered as a hepatic damage marker. Blood biomarkers are commonly employed in large-scale general population-based epidemiologic research since liver biopsies are extremely hard to obtain. Additionally, liver enzymes can cause significant rises in blood liver enzyme levels, even if they are secreted by a small fraction of injured hepatocytes. Consequently, these enzymes should be regarded as markers of liver damage, according to the clinical recommendations established by the American College of Gastroenterology (ACG) [33, 34]. ALT, AST, ALP and GGT have often been employed in prior research as indicators for assessing liver damage [35–38]. The elevation of AST and ALT in serum is due to the leakage of intracellular enzymes into the blood after hepatocyte damage. ALP is mainly derived from the liver, and the elevation of ALP indicates hepatocyte damage, bile secretion obstruction, or biliary obstruction. GGT mainly exists in hepatocyte membranes and microsomes and is widely distributed in capillary bile ducts on one side of the hepatocyte and in the whole biliary system, and the level of GGT rises in serum when liver injury occurs [39].

In the present study, a certain correlation was found between SII and markers of liver injury, and SII was negatively correlated with ALT, AST and GGT. There are several considerations to be highlighted: 1) ALT which is mainly found in hepatocytes is considered a more specific indicator of liver disease, but the detection rate of ALT abnormalities is not high in alcohol-related liver disease and some cases of autoimmune hepatitis (AIH) [13]; 2) AST mainly exists in mitochondria, and when in the setting of acute viral hepatitis, the hepatocyte mitochondria are still preserved intact despite hepatocyte damage, which may lead to biased blood AST values and elevated SII; 3) The most common causes of elevated GGT contain obesity and excessive alcohol consumption, or it may be induced by medications. Although elevated GGT is less specific for liver disease, it is one of the optimal predictors of liver death. The results of this research showed that when SII exceeded 818.40 (1,000 cells/μl), ALT, AST, ALP and GGT basically all displayed an increasing trend, demonstrating the correlation between SII and markers of liver impairment. In addition, SII can be calculated from routine blood data, which is simple, easy and reproducible, and can reduce the cost of medical testing for patients to a certain extent. Among subgroup analyses, the interaction tests implied that the correlation between SII and markers of liver injury was not dependent on gender, age and BMI (all p for interaction>0.05), suggesting that these connections remain true across various demographic contexts.

There are several advantages to this survey. The validity and representativeness of this study were improved by the high sample size and adequate covariate adjustment. Furthermore, the big sample size ensured that subgroup analyses could be conducted. False positives are less likely when sensitivity analysis is performed. Nevertheless, this research still exposes several limits. Due to being unable to establish causation based on the cross-sectional study design, large samples from prospective studies are required to clarify causality. Besides, there are other confounders, such as a history of long-term use of medications like hepatoprotective and antiviral drugs, which may still have an impact on outcomes in spite of control for some confounders in this study. These factors, however, have not been recorded in NHANES, making them unavailable for this study. Additionally, the interactions between inflammation and indicators of liver injury are complex. Therefore, it may be inappropriate to generalize the findings of this study.

## Conclusion

The research findings manifested the presence of a negative correlation between SII and ALT, AST and GGT, and a positive correlation with ALP. However, larger prospective investigations are greatly needed to confirm the findings.

## Supporting information

S1 Checklist STROBE statement—Checklist of items that should be included in reports of observational studies. (DOC)

S1 Data (XLS)

S1 File (DOCX)
