# Supplementary material for: Association between SII and markers of liver injury: A cross-sectional study from the NHANES (2017–2020)
Source: PLoS One. 2024 Jul 25;19(7):e0303398. doi: 10.1371/journal.pone.0303398 (PMC11271860; doi:10.1371/journal.pone.0303398)
Supplement: S1 File — (DOCX) [file pone.0303398.s003.docx]

DOI：10.6084/m9.figshare.25594677

https://figshare.com/s/e64f08b4d2cd018657e8
